# Differential Mitochondrial Gene Expression in Adipose Tissue Following Weight Loss Induced by Diet or Bariatric Surgery

**DOI:** 10.1210/clinem/dgab072

**Published:** 2021-02-09

**Authors:** Birgitta W van der Kolk, Maheswary Muniandy, Dorota Kaminska, Marcus Alvarez, Arthur Ko, Zong Miao, Armand Valsesia, Dominique Langin, Maija Vaittinen, Mirva Pääkkönen, Riikka Jokinen, Sanna Kaye, Sini Heinonen, Kirsi A Virtanen, Daniel P Andersson, Ville Männistö, Wim H Saris, Arne Astrup, Mikael Rydén, Ellen E Blaak, Päivi Pajukanta, Jussi Pihlajamäki, Kirsi H Pietiläinen

**Affiliations:** 1 Obesity Research Unit, Research Program for Clinical and Molecular Metabolism, Faculty of Medicine, University of Helsinki, Finland; 2 Institute of Public Health and Clinical Nutrition, University of Eastern Finland, Kuopio, Finland; 3 Department of Human Genetics, David Geffen School of Medicine at UCLA, Los Angeles, CA, USA; 4 Department of Medicine, David Geffen School of Medicine at UCLA, Los Angeles, CA, USA; 5 Bioinformatics Interdepartmental Program, UCLA, Los Angeles, CA, USA; 6 Nestlé Institute of Health Sciences, 1015 Lausanne, Switzerland; 7 Institut National de la Santé et de la Recherche Médicale (Inserm), Université Paul Sabatier, Institute of Metabolic and Cardiovascular Diseases, Toulouse, France; 8 Department of Biochemistry, Toulouse University Hospitals, France; 9 Turku Bioscience Centre, University of Turku and Åbo Akademi University, Finland; 10 Department of Medicine, Endocrinology and Clinical Nutrition, Kuopio University Hospital, Kuopio, Finland; 11 Turku PET Center, Turku University Hospital, Turku, Finland; 12 Department of Medicine (H7), Karolinska Institutet, Karolinska University Hospital, Huddinge, Stockholm, Sweden; 13 Department of Medicine, University of Eastern Finland and Kuopio University Hospital, Kuopio, Finland; 14 Department of Human Biology, NUTRIM School of Nutrition and Translational Research in Metabolism, Maastricht University, MD Maastricht, The Netherlands; 15 Department of Nutrition, Exercise and Sports, Faculty of Science, University of Copenhagen, Copenhagen, Denmark; 16 Institute for Precision Health, David Geffen School of Medicine at UCLA, Los Angeles, CA, USA; 17 Obesity Center, Abdominal center, Helsinki University Hospital and University of Helsinki, Helsinki, Finland

**Keywords:** adipose tissue, mitochondria, weight loss, diet-induced, bariatric surgery, transcriptomics

## Abstract

**Context:**

Mitochondria are essential for cellular energy homeostasis, yet their role in subcutaneous adipose tissue (SAT) during different types of weight-loss interventions remains unknown.

**Objective:**

To investigate how SAT mitochondria change following diet-induced and bariatric surgery–induced weight-loss interventions in 4 independent weight-loss studies.

**Methods:**

The DiOGenes study is a European multicenter dietary intervention with an 8-week low caloric diet (LCD; 800 kcal/d; n = 261) and 6-month weight-maintenance (n = 121) period. The Kuopio Obesity Surgery study (KOBS) is a Roux-en-Y gastric bypass (RYGB) surgery study (n = 172) with a 1-year follow-up. We associated weight-loss percentage with global and 2210 mitochondria-related RNA transcripts in linear regression analysis adjusted for age and sex. We repeated these analyses in 2 studies. The Finnish CRYO study has a 6-week LCD (800-1000 kcal/d; n = 19) and a 10.5-month follow-up. The Swedish DEOSH study is a RYGB surgery study with a 2-year (n = 49) and 5-year (n = 37) follow-up.

**Results:**

Diet-induced weight loss led to a significant transcriptional downregulation of oxidative phosphorylation (DiOGenes; ingenuity pathway analysis [IPA] z-scores: −8.7 following LCD, −4.4 following weight maintenance; CRYO: IPA z-score: −5.6, all *P* < 0.001), while upregulation followed surgery-induced weight loss (KOBS: IPA z-score: 1.8, *P* < 0.001; in DEOSH: IPA z-scores: 4.0 following 2 years, 0.0 following 5 years). We confirmed an upregulated oxidative phosphorylation at the proteomics level following surgery (IPA z-score: 3.2, *P* < 0.001).

**Conclusions:**

Differentially regulated SAT mitochondria-related gene expressions suggest qualitative alterations between weight-loss interventions, providing insights into the potential molecular mechanistic targets for weight-loss success.

Obesity, one of the largest public health threats globally (1), associates with various debilitating diseases, including type 2 diabetes (T2DM), cardiovascular disorders, and some cancers (2, 3).

Weight loss effectively reduces health risks among people who are overweight or with obesity. A moderate body weight reduction of 5% to 10% induced through dieting significantly reduces disease risk and improves the metabolic profile (4). However, long-term weight maintenance resulting from lifestyle interventions remains rather poor (5). Bariatric surgery, while more invasive, leads to greater weight losses and more often to T2DM remission compared with diet-induced weight loss (6, 7). Reductions in body weight are predominantly due to the loss of adipose tissue, although the underlying processes responsible for adipose tissue function during weight loss and maintenance following different types of interventions are poorly understood.

Mitochondrial function in subcutaneous adipose tissue (SAT) may be an important contributor responding to energy balance changes following weight loss. SAT mitochondrial oxidative metabolism is diminished in obesity (8-10)—a phenomenon that likely associates with both the persistence of the obesity phenotype and the development of obesity-related metabolic diseases (11). Previously, we, and others, reported a downregulation of mitochondria-related pathways in diet-induced weight loss and maintenance (12, 13), with findings that remain inconsistent (14, 15). In contrast, an increased SAT mitochondrial capacity was observed following surgically induced weight loss (16-18), indicating that bariatric surgery may cause a metabolically more advantageous change to the SAT mitochondrial function than dieting. This implies that SAT mitochondrial activity could impact the mechanism explaining more successful weight maintenance following different intervention types, while the true long-term effects are unclear.

Furthermore, weight-loss interventions are generally difficult to reconcile and one contributing factor stems from differences in the degree of weight loss achieved following an intervention. Studies focusing on the comprehensive characterization of SAT mitochondria, using standardized analysis pipelines for different types of long-term weight-loss interventions, are lacking. Here, we aimed to retrospectively investigate mitochondria-related RNA expression profiles in SAT following 2 diet-induced and 2 bariatric surgery–induced weight-loss interventions. We performed a standardized set of bioinformatics analyses, considering the percentage weight loss across all study cohorts. In addition, we analyzed the SAT proteome following surgery-induced weight loss.

## Research Design and Methods

### Study Designs

The Diet, Obesity and Genes (DiOGenes) study is a multicenter, randomized, dietary intervention study (ClinicalTrials.gov ID number NCT00390637) (19). Briefly, 938 healthy adults with overweight or obesity (body mass index [BMI] 27-45 kg/m^2^) without concomitant medications followed a low-calorie diet (LCD; 800 kcal/day) for 2 months. Participants who lost ≥8% of their body weight were subsequently assigned to 1 of 5 diets (a 2 × 2 factorial combination diet of low/high protein and a low/high glycemic index or a control diet) in the 6-month weight-maintenance phase. Altogether, 548 participants completed both phases. Here, we examined data from 314 participants, for whom abdominal SAT RNA sequencing data from at least 2 time points were available. Weight and metabolic health were assessed at baseline, 2 months, and 8 months.

The Kuopio OBesity Surgery (KOBS) is a prospective observational study investigating the metabolic consequences of obesity surgery (20). The study criteria were (1) a BMI ≥ 40 kg/m^2^, or BMI ≥ 35 kg/m^2^ with an obesity-associated comorbidity; (2) a failure of traditional obesity treatments; and (3) no other surgical contraindications. Here, we report analyses from 172 participants (34% with T2DM) who underwent Roux-en-Y gastric bypass (RYGB) surgery and for whom abdominal SAT RNA sequencing data were available at baseline and at 12 months follow-up. Additionally, we studied SAT proteomics in a subset of 29 individuals without T2DM, respectively. Weight and metabolic health were assessed at baseline and 12 months.

CRYO is a case-control dietary intervention study (ClinicalTrials.gov ID NCT01312090) (12, 21, 22), utilized to replicate our findings on diet-induced weight loss. Briefly, 19 adults with obesity and no concomitant medications lost weight through a 6-week modified LCD (800-1000 kcal/day), followed by a weight-maintenance phase with daily reductions of 500 to 1000 kcal from their pre-LCD diet until 12 months. SAT microarrays, weight, and metabolic health were assessed at baseline, 5 months, and 12 months.

To replicate our findings on surgery-induced weight loss, we utilized the Effects of Different Surgical Methods to Treat Obesity (DEOSH) study (ClinicalTrials.gov ID NCT01785134). This study (23) consisted of 82 females with morbid obesity (BMI ≥ 40 kg/m^2^) or BMI ≥ 35 kg/m^2^ with obesity-related comorbidities who underwent RYGB surgery. Here, we analyzed data for 49 individuals for whom abdominal SAT microarray data were available, together with assessment of weight and metabolic health at baseline and 2 and 5 years.

The ethical approvals were obtained from the ethics committees of each center/country for the multicenter DiOGenes cohort, from the Northern Savo Hospital District, Kuopio, Finland for KOBS, from the hospital districts Helsinki and Uusimaa, as well as Southwest Finland, Finland for CRYO, and from the Karolinska Institutet in Stockholm, Sweden for the DEOSH study. All studies adhered to the Helsinki Declaration regarding participants’ informed consent.

### Adipose Tissue Biopsy, RNA Preparation, and Transcriptomics Analyses

In all studies, SAT biopsy specimens were snap-frozen in liquid nitrogen and stored at −80 °C until further analysis. In DiOGenes, after an overnight fast, abdominal SAT needle biopsies were collected 6 to 8 cm laterally from the umbilicus under local anesthesia. Total RNA was extracted and analyzed as described elsewhere (24, 25). For each sample, gene expression was then examined using 100-nucleotide-long paired-end RNA sequencing with an Illumina HiSeq 2000 of libraries prepared using the Illumina TruSeq kit. We used GenomicAlignments to retrieve the number of reads mapping onto 53 343 genes (GRCh37 assembly). Only reads with both ends mapping onto a single gene were considered (24, 25).

In KOBS, abdominal SAT baseline samples involved open biopsies taken during surgery, while 12-month postsurgical samples were collected as open biopsies under local anesthesia. Total RNA was extracted and purified using the miRNeasy Mini Kit. RNA sequencing libraries were constructed with the TruSeq Stranded mRNA Library Prep Kit and underwent 69-nucleotide-long paired-end sequencing on the Illumina HiSeq 4000 machine at a depth of 40 to 50 M reads per sample. The Rsubread R package was used to count all reads mapping onto a final list of 15 014 genes (GRCh38 assembly). Only reads with both ends mapping onto a single gene were considered.

In CRYO, periumbilical SAT biopsies were obtained surgically under local anesthesia after an overnight fast. Total RNA was extracted and the global transcript profiles of SAT were analyzed using Affymetrix HG-U133 Plus 2 microarrays (12).

In DEOSH, abdominal SAT biopsy specimens were obtained from a surgical incision at baseline and at follow-up. Total RNA was extracted and global transcript profiles of SAT were analyzed using ClariomTM D arrays (Affymetrix, Santa Clara, CA, USA) (26).

### SAT Protein Isolation, LC-ESI-MS/MS Preparation, and Proteome Analysis in KOBS

Proteins were extracted from the SAT specimens by homogenizing the tissue with a TissueLyser LT instrument (QIAGEN) in a lysis buffer containing broad-range protease and phosphatase inhibitors. We collected the protein fraction under the lipid layer and determined the concentrations. Because the SAT specimens contained blood contaminants, we depleted the high-abundant serum proteins and again determined concentrations. Next, we performed in-solution digestion with trypsin, sodium deoxycholate removal, desalting, and peptide elution, after which equal amounts of total protein were prepared. Liquid chromatography electrospray ionization tandem mass spectrometric (LC-ESI-MS/MS) analysis was conducted on a nano-high-performance liquid chromatography (HPLC) system (Easy-nLC1200) coupled to the Q Exactive HF mass spectrometer (Thermo Fisher Scientific, Bremen, Germany) equipped with a nano-electrospray ionization source using the data-dependent operation mode.

### Statistical Analyses

#### Anthropometric and clinical variables

Changes in anthropometric and clinical parameters before and after interventions were analyzed using a mixed-model ANOVA (DiOGenes, CRYO and DEOSH) applying the restricted maximum likelihood (REML) method (27). The participant ID was used as a random factor, selecting the unstructured covariance structure. The Bonferroni post hoc correction was used for comparisons between timepoints. In KOBS, changes in anthropometric and clinical variables were analyzed using paired samples t-tests, in which skewed variables were log_e_-transformed before analysis. We considered p < 0.05 statistically significant.

#### Differential RNA expression analyses

The DiOGenes dietary intervention and the KOBS bariatric surgery intervention were used as the discovery material, while we used the CRYO and DEOSH cohorts as the diet- and surgery-induced replication datasets, respectively.

First, to determine the effects of weight loss on the global SAT transcriptomics profiles of the DiOGenes and KOBS cohorts, we performed differential expression analysis using the linear model (package limma and limma-voom in R-Bioconductor (28)). We identified the altered transcripts that associated with weight-loss percentage (%) within each participant (from before and after intervention). In both cohorts, we adjusted the regression model for sex and age, and in DiOGenes, additionally for study center and sequencing center. In KOBS, we further adjusted the model for technical covariates. We corrected *P* values for multiple testing (Benjamini and Hochberg method (29)) and considered a false-discovery rate (FDR) *P* < 0.05 statistically significant.

Second, we performed a targeted differential expression analysis on transcripts affecting mitochondria, based on 2210 genes selected from the MitoMiner 4.0 database (30). We applied the same statistical models as above for the mitochondria-targeted analyses for DiOGenes and KOBS, and then repeated them in the CRYO and DEOSH datasets. We identified the significantly altered gene expressions (CRYO: FDR *P* < 0.05; DEOSH nominal *P* < 0.05) that associated with weight-loss percentage, adjusted for sex (CRYO) and age (CRYO and DEOSH).

Next, we identified individuals with 15% to 20% weight loss in DiOGenes and KOBS and repeated the targeted mitochondrial differential expression analysis using the same statistical models as above.

#### SAT proteomics data normalization and differential expression analysis

We analyzed the effects of weight loss on the SAT proteomics in a subset (n = 29) of the KOBS cohort. Data pre-processing included the removal of blood proteins (31) and resulted in 1204 identified proteins, for which we performed differential expression analysis using a linear model (package limma in R-Bioconductor). Significantly changed protein expressions (nominal *P* < 0.1) that associated with weight-loss percentage within each participant were identified and adjusted for sex and age.

#### Biological pathway analyses

We further investigated significantly changed genes and proteins identified from the differential analyses using the ingenuity pathway analysis tool (IPA) (August 2019, Ingenuity Systems, Redwood City, CA, USA).

#### Associations between mitochondria-related gene expression and clinical variables in DiOGenes and KOBS

We calculated standardized beta coefficients between the delta (postintervention minus baseline) oxidative phosphorylation (OXPHOS) pathway score and the delta clinical outcome measures using linear regression analysis (model 1). The OXPHOS score was calculated by averaging, for each person, the z-scores of the 102 genes in the OXPHOS pathway. In model 2, we added sex and age (including study center and sequencing center for DiOGenes) as covariates. Skewed clinical variables were log_e_-transformed. In model 3, a dimension reduction was performed on the gene expressions in the OXPHOS pathway using principal component analysis (PCA). We performed a linear regression adjusted for sex and age (including study center and sequencing center for DiOGenes) using normalized gene expression values, retaining only enough principal components (PCs) (3 in DiOGenes and 2 in KOBS) to account for 80% of the total variances captured. We considered *P* < 0.05 statistically significant.

All data were analyzed using SPSS for Mac (version 24.0; SPSS Inc., Chicago, IL, USA) or R statistical programming language (version 3.3.3).

## Results

### Participant Characteristics and Weight Loss

The DiOGenes dietary intervention and the KOBS bariatric surgery intervention were used as the discovery material, while we used the CRYO and DEOSH cohorts, respectively, as diet- and surgery-induced replication datasets. Table 1 summarizes the anthropometric and clinical characteristics of the discovery studies, while Supplemental Table 1 (32) provides the characteristics of the replication studies. Across all cohorts, participants lost significant amounts of weight (*P* < 0.001). The clinical measures for lipid metabolism and insulin sensitivity primarily reflected changes in weight and were ameliorated in all studies (Table 1 and Supplemental Table 1 (32)).

**Table 1.** Participant Characteristics in the Diet-Induced Weight Loss DiOGenes Cohort and the Surgery-Induced Weight Loss KOBS Cohort

	DiOGenes baseline	DiOGenes 2 months	DiOGenes 8 months	DiOGenes *P*	KOBS baseline	KOBS 12 months	KOBS *P*
Female sex (%)	64.5%				72%		
Age, y	42.8 ± 6.6				48.3 ± 9.3		
Weight loss (%)	–	11.2% ± 2.8%	11.9% ± 7.1%		–	22.4% ± 7.7%	
Weight, kg	98.2 ± 15.8	87.1 ± 13.6*	86.3 ± 14.6*	<0.001	121.6 ± 17.3	94.5 ± 16.5	<0.001
BMI, kg/m^2^	34.2 ± 4.3	30.4 ± 3.9*	30.1 ± 4.2*	<0.001	43.0 ± 5.2	33.3 ± 5.0	<0.001
Cholesterol, mmol/L	5.0 ± 0.8	4.3 ± 0.7*	5.0 ± 0.9^#^	<0.001	4.0 (3.5-4.7)	4.4 (3.8-4.9)	<0.001
LDL, mmol/L	3.1 ± 0.8	2.6 ± 0.7*	3.0 ± 0.8^#^	<0.001	2.30 ± 0.80	2.39 ± 0.79	0.184
HDL, mmol/L	1.3 ± 0.3	1.2 ± 0.3*	1.4 ± 0.3* ^#^	<0.001	1.1 (0.9-1.3)	1.5 (1.3-1.8)	<0.001
NEFA, µmol/L	680 ± 340	709 ± 232	568 ± 247* ^#^	<0.001	700 (550-845)	480 (350-605)	<0.001
TAG, mmol/L	1.2 (0.9-1.6)	0.9 (0.8-1.3)*	1.1 (0.8-1.5)^#^	0.002	1.3 (1.1-1.9)	1.1 (0.8-1.4)	0.003
Glucose, mmol/L	5.1 ± 0.7	4.8 ± 0.5*	4.9 ± 0.5*	<0.001	6.1 (5.4-6.8)	5.4 (5.0-5.9)	<0.001
Insulin, mU/L	8.6 (6.2-12.8)	6.3 (4.1-9.5)*	7.4 (5.0-9.9)* ^#^	<0.001	16.0 (10.5-21.8)	6.5 (4.2-11.0)	<0.001
HOMA-IR	2.0 (1.2-2.8)	1.3 (0.9-1.8)*	1.6 (1.1-2.3)* ^#^	<0.001	4.3 (2.7-6.6)	1.7 (1.1-2.9)	<0.001

Data are shown as mean ± SD (normally distributed variables), median (interquartile range, for skewed variables), or proportion (%, for categorical variables). In DiOGenes, *P* values were obtained using a mixed-model ANOVA using the restricted maximum likelihood (REML) method to examine the effects of time on anthropometric and clinical parameters before and after interventions among 121 individuals. The participant ID was used as a random factor and we used an unstructured covariance structure. The Bonferroni was used for post hoc corrections between timepoints: *P* < 0.05 *different from baseline, ^#^different from the 2-month time point. In KOBS, *P* values were obtained using the paired *t-*tests for 172 individuals. Skewed variables were log_e_-transformed before analysis. Abbreviations: BMI, body mass index; HDL, high-density lipoprotein; HOMA-IR, homeostatic model for the assessment of insulin resistance; LDL, low-density lipoprotein; NEFA, nonesterified fatty acid; TAG, triacylglycerol.

In DiOGenes, average weight loss reached 11.2% ± 2.8% following LCD and 11.9% ± 7.1% following the weight-maintenance period (Table 1). In CRYO, participants lost on average 11.5% ± 5.3% weight after the 5-month and 9.0% ± 7.2% after the 12-month period (Supplemental Table 1 (32)). In KOBS, average weight loss reached 22.4% ± 7.7% at 12 months after surgery (Table 1), while in DEOSH, average weight loss was 31.6% ± 9.3% at 2 years and 27.5% ± 10.4% at 5 years after surgery (Supplemental Table 1 (32)).

### SAT Mitochondrial Gene Pathways Changed in Opposing Directions in Global Transcriptomics Analyses

We first explored changes in the global transcriptomics patterns in SAT using regression analysis against the weight-loss percentage in DiOGenes and KOBS. In DiOGenes, following the 2-month LCD phase, we identified 11 488 significant associations (FDR *P* < 0.05) between the weight-loss percentage and gene transcripts; among these, 1378 (12%) were mitochondria-related (Supplemental Table 2 (32)). Following the weight-maintenance phase (8 months), we identified 9622 associations, of which 11% were mitochondria-related (Supplemental Table 2 (32)). In KOBS, we identified 8756 associations between the weight-loss percentage and gene transcripts at baseline and 12 months after surgery, of which 1197 (14%) were mitochondria-related (Supplemental Table 3 (32)).

**Figure 1.**
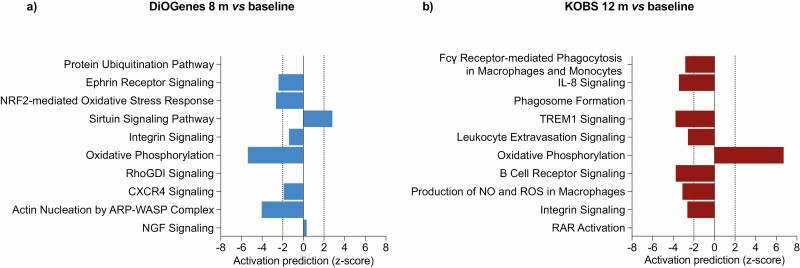
Gene enrichment for biological pathways using significantly differentially expressed global genes associated with the weight-loss percentage following the diet-induced weight-loss DiOGenes study (n = 121) (A) and the surgery-induced weight-loss KOBS study (n = 171) (B). The top 10 significant pathways from the ingenuity pathway analysis (IPA) tool (*P* < 0.001) for both cohorts are presented. For several pathways, IPA provided z-scores for the pathway directionality by calculating the observed number of “activated” genes (z-score > 0), “inhibited” genes (z-score < 0), or no directionality (z-score = 0). Z-scores > 2 or < −2 were considered statistically significant. Results are ranked according to statistical significance.

To gain insight into the biological pathways, we used IPA on the top (FDR *P*-sorted) 2000 differentially expressed transcripts associated with weight-loss percentage. Interestingly, both weight-loss interventions showed significant differences in the mitochondrial gene–related pathways (Fig. 1). The OXPHOS pathway was among the top 10 (using the Fisher’s exact *P*) significantly enriched pathways in both cohorts, resulting in opposing activation prediction scores (DiOGenes z-score −5.4 vs KOBS z-score 6.7). Tricarboxylic acid cycle (TCA) cycle II, sirtuin signaling, and the NRF2-mediated oxidative stress response were also significantly changed after both diet- and surgery-induced weight loss (data not shown).

### Analyses of Differentially Expressed Mitochondria-Related Genes

We subsequently focused on 2210 mitochondria-targeting genes by extracting them from the MitoMiner database (30). Using FDR *P* < 0.05 as the cutoff, we found that the weight-loss percentage following the LCD phase in the DiOGenes cohort significantly associated with a change in 1437 transcripts, 65% of which were downregulated (Supplemental Table 4 (32)). Between baseline and 8 months, we found 1173 associations, 61% of which were downregulated. By contrast, in KOBS, we identified 1194 significant associations, mostly involving upregulated transcripts (70%) following weight loss between baseline and 12 months (Supplemental Table 4 (32)). Additionally, in KOBS, we stratified the cohort for T2DM status. The direction of mitochondria-related transcripts remained similar, although the statistical power was diminished in the smaller subgroups (Supplemental Table 5 (32)). These results indicate that the majority of transcripts targeting mitochondria were affected by the weight-loss percentage in DiOGenes and KOBS, although in opposing directions.

Next, we used the CRYO (diet-induced) and DEOSH (surgery-induced) cohorts to reproduce the mitochondrial results in SAT, finding similar patterns as those observed in DiOGenes and KOBS. Although these replication studies were smaller in size, both studies had longer follow-up periods than DiOGenes and KOBS. In CRYO, we found that most (75%) of the 581 associations between the weight-loss percentage and transcripts were downregulated (Supplemental Table 6 (32)). In contrast, in DEOSH, most of the significant (nominal *P* < 0.05) transcripts were upregulated following weight loss both between baseline and 24 months (96% of 483 transcripts) and between baseline and 5 years (81% of 120 transcripts) (Supplemental Table 7 (32)).

### OXPHOS Pathway Downregulated Following Diet-Induced, but Upregulated Following Surgery-Induced Weight Loss

**Figure 2.**
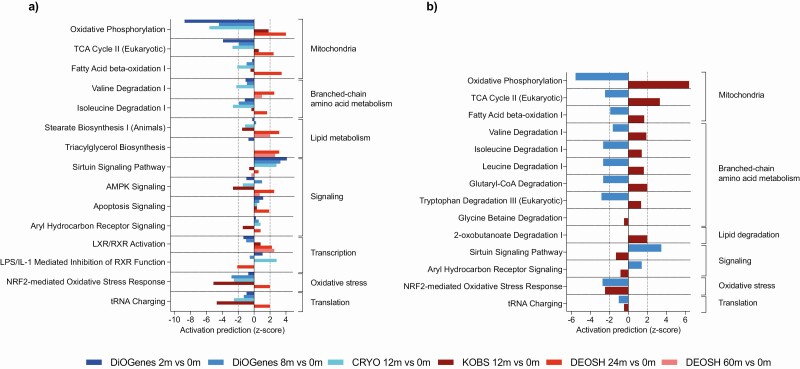
Gene enrichment for biological pathways using significantly differentially expressed mitochondria-related genes associated with the weight-loss percentage following diet- and surgery-induced weight loss across all studies (in total, 6 postintervention vs baseline comparisons) (A) and in groups matched for 15% to 20% weight loss from the diet-induced weight-loss DiOGenes study (n = 24) and surgery-induced weight-loss KOBS study (n = 34) (B). In (A), we selected the top 10 significant pathways from the ingenuity pathway analysis (IPA) tool (*P* < 0.001) from each comparison; the pathway results are shown when present in at least 2 of 6 of the top 10 selections. In (B), the results are presented for the top 10 significant pathways from the IPA tool (*P* < 0.001) from both studies. For several pathways, IPA provided z-scores for the pathway directionality by calculating the observed number of “activated” genes (z-score > 0), “inhibited” genes (z-score < 0), or no directionality (z-score = 0). Z-scores > 2 or < −2 were considered significant. Results are ranked according to biological function. In (A), gradients indicated by blue bars (from dark to light blue) indicate diet-induced weight-loss comparisons: DiOGenes 2 months vs baseline (n = 261); DiOGenes 8 months *vs* baseline (n = 121); and CRYO 12 months vs baseline (n = 19). Gradients indicated by red bars (from dark to light red) indicate the surgery-induced weight-loss studies: KOBS, 12 months vs baseline (n = 171); DEOSH, 24 months vs baseline (n = 49); and DEOSH, 60 months vs baseline (n = 37). Abbreviation: m, months.

At the pathway level, we found a strong downregulation of the OXPHOS pathway in the dietary-induced DiOGenes cohort (z-scores: −8.7 at 2 months vs baseline and −4.4 at 8 months vs baseline), but an upregulation in the surgery-induced KOBS cohort (z-score: 1.8). These findings persisted in the CRYO cohort (z-score: −5.6) and the DEOSH cohort (z-score: 4.0 for 24 months vs baseline), but not 60 months vs baseline. The top 10 (sorted based on the Fisher’s exact *P*) IPA pathways, for which a pathway emerged in at least 2 comparisons, appear in Fig. 2a.

**Figure 3.**
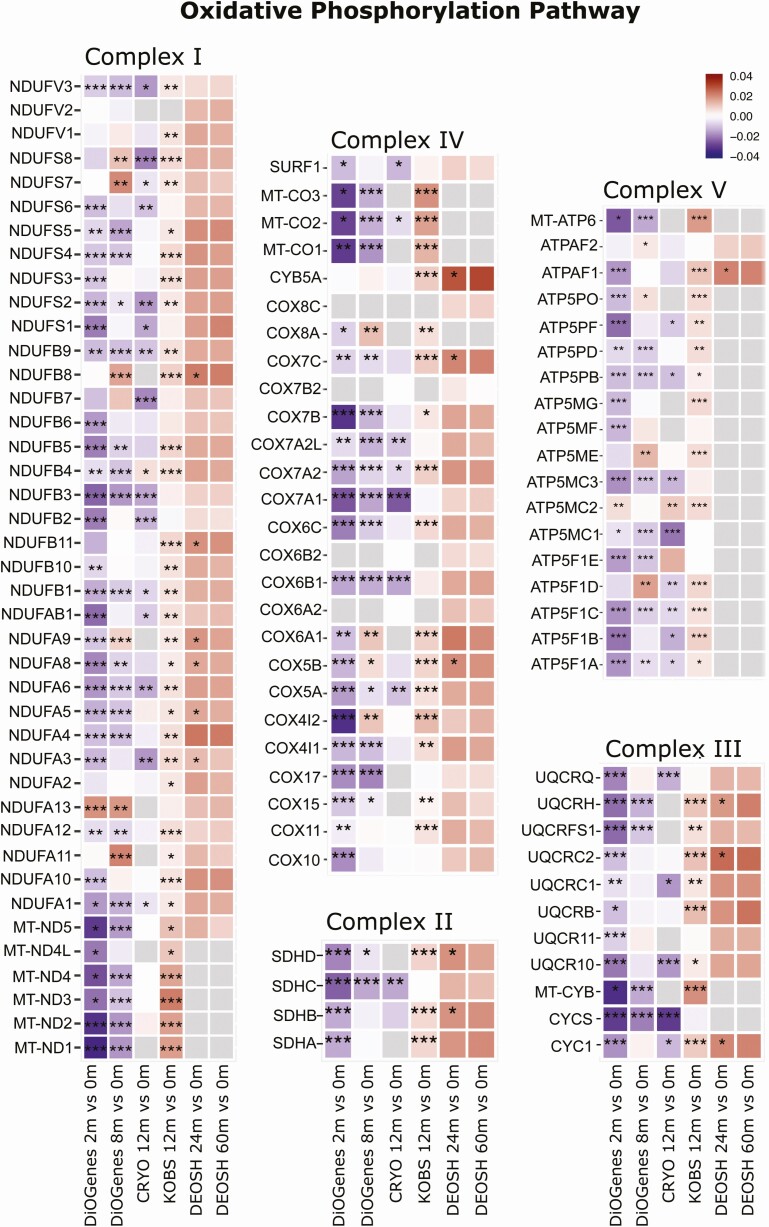
Heat map showing the differential expression of the individual genes in the oxidative phosphorylation (OXPHOS) pathway associated with weight-loss percentage following diet- and surgery-induced weight loss. Log_2_-fold changes indicated for the 5 primary complexes of the electron transport chain genes. The color in the heat maps reflect the differential expressions associated with the weight-loss percentage before and after weight loss, where red indicates up- and blue indicates downregulation. Asterisks indicate statistically significant differential expressions: ***P < 0.001; **P < 0.01; *P < 0.05. Gray squares indicate unmeasured transcripts. The 3 left-most columns show the diet-induced weight-loss comparisons: DiOGenes, 2 months vs baseline (n = 261); DiOGenes, 8 months vs baseline (n = 121); and CRYO, 12 months vs baseline (n = 19). The 3 right-most columns show the surgery-induced weight-loss studies: KOBS, 12 months vs baseline (n = 171); DEOSH, 24 months vs baseline (n = 49); and DEOSH, 60 months vs baseline (n = 37). Abbreviation: m, months.

Since the OXPHOS pathway was significantly differently regulated by the 2 weight-loss intervention types, we next examined the individual transcripts for that pathway across the 4 respective cohorts. The overall pattern clearly reveals a downregulation of the transcripts in the dietary-induced interventions (DiOGenes and CRYO) and an upregulation in the surgery-induced interventions (KOBS and DEOSH), suggesting opposing effects on the mitochondrial respiratory machinery (Fig. 3).

Genes encoding the 5 OXPHOS complexes originate both from the nucleus and mitochondria. To this end, it is noteworthy that the majority of the top 10 most downregulated transcripts in DiOGenes and the majority of the top 10 most upregulated transcripts in KOBS in the OXPHOS pathway were encoded by mitochondrial DNA (mtDNA) (based on fold changes) (Supplemental Table 4 (32)). In both studies, *MT-ND1*, *MT-ND2,* producing complex I NADH dehydrogenase subunits, and *MT-CO1,* the primary subunit of the cytochrome c oxidase (IV) complex, emerged as the most differentially expressed mitochondria-encoded transcripts.

### Other Enriched Mitochondrial-Related Pathways in Diet- and Surgery-Induced Weight Loss

The TCA cycle II, fatty acid β-oxidation I, and branched-chain amino acid (BCAA) degradation pathways were also significantly changed following weight loss (Fig. 2a). We observed a downregulation of mitochondria-related pathways specifically in all diet-induced weight-loss cohorts, where the TCA cycle pathway represented the second most predicted downregulated pathway (z-scores: < 2). The mitochondria-related pathways in the surgery-induced cohorts varied more in their responses than the diet-induced cohorts (Fig. 2a). Nevertheless, the activation prediction score for these mitochondria-related pathways consistently indicated a downregulation for diet-induced and an upregulation for surgery-induced weight loss.

### Consistently Opposing Mitochondria-Related Patterns Among Groups Matched for Weight-Loss Percentage

Although the results above were derived from analyses examining weight-loss percentage, we further addressed the possibility that our findings resulted from the larger amount of weight loss in bariatric surgery interventions. To this end, we identified individuals with similar weight-loss percentage (15%-20%) at the end of follow-up among the DiOGenes and KOBS cohorts.

We compared the mitochondria-related gene expression differences associated with weight-loss percentage at 8 months (DiOGenes: n = 24, 58% female, mean age: 43 ± 7 years, mean weight loss: 17.2 ± 1.5%) and at 12 months (KOBS: n = 34, 65% female, mean age: 49 ± 7 years, mean weight loss: 17.4 ± 1.4%) vs baseline, respectively. Based on the FDR *P* < 0.05 cutoff, we identified 657 associations between weight-loss percentage and changes in transcript expression, most (74%) of which were downregulated in DiOGenes. In KOBS, we found 270 associations, most (79%) of which were upregulated (Supplemental Table 8 (32)). At the pathway level, we again found a downregulation of OXPHOS transcripts following diet-induced weight loss (z-score: −5.6) and an upregulation following surgery-induced weight loss (z-score: 6.4) (Fig. 2b). Moreover, the TCA cycle II pathway also significantly changed in opposing directions (z-score: >|2|); the fatty acid β-oxidation I and BCAA degradation pathways changed in opposing directions, although not statistically significantly. This consistency in results suggests that our findings did not result from weight-loss differences related to the interventions.

### SAT Mitochondria-Related Proteins Upregulate Following Surgical Intervention

**Figure 4.**
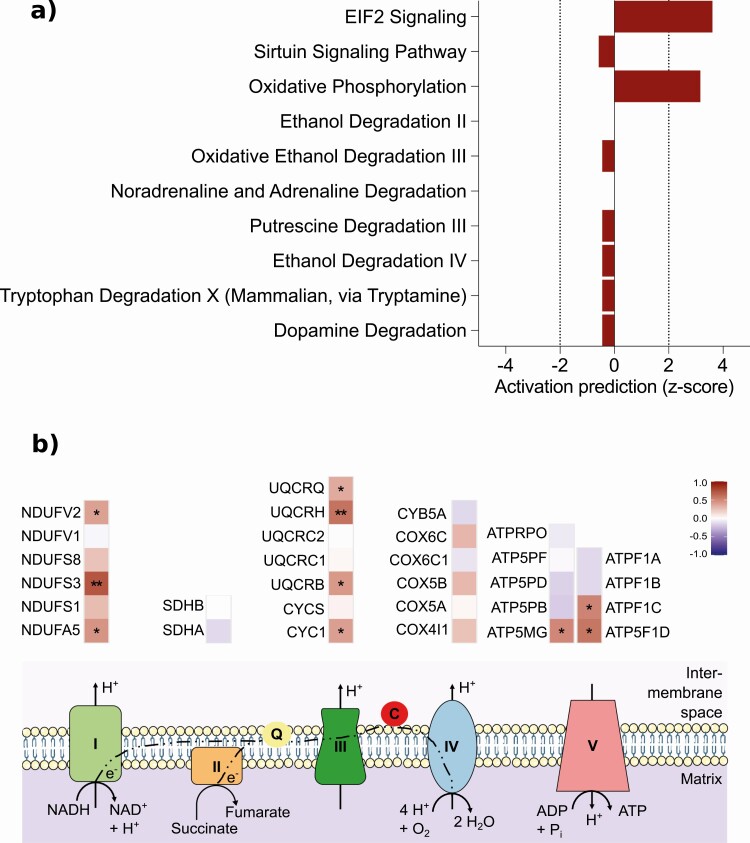
SAT mitochondria-related proteins were upregulated following surgical intervention. Protein enrichment for biological pathways using differentially expressed proteins (nominal *P* < 0.1) associated with the weight-loss percentage following surgery-induced weight loss in KOBS (n = 29) (A), the top 10 significant findings from the ingenuity pathway analysis (IPA) tool (*P* < 0.001) are shown. For several pathways, IPA provided z-scores for pathway directionality by calculating the observed number of “activated” genes (z-score > 0), “inhibited” genes (z-score < 0), or no directionality prediction (z-score = 0). Z-scores > 2 or < −2 were considered statistically significant. Results ranked according to statistical significance. In (B), a heat map showing the differential expression of the individual proteins in the oxidative phosphorylation (OXPHOS) pathway associated with weight-loss percentage following surgery-induced weight loss. Log_2_-fold changes indicated for the 5 primary complexes of the electron transport chain proteins. Only measured proteins are shown. The color in the heat maps reflect the differential expressions associated with the weight-loss percentage before and after weight loss, where red indicates up- and blue indicates downregulation. Asterisks indicate statistically significant differential expressions: **P < 0.05; *P < 0.1. Abbreviations: c, cytochrome c; q, ubiquinone.

To determine if the transcriptomics results following surgery-induced weight loss remained consistent at other levels, we examined the SAT proteome in 29 individuals from the KOBS cohort. We analyzed weight-loss percentage with the protein expression postoperatively, finding that 284 of 1204 identified SAT proteins expressed significantly differently (nominal *P* < 0.1, Supplemental Table 9 (32)), 95% of which were upregulated. Moreover, 101 (30%) of the significantly changed proteins were mitochondria-related, all upregulated. Likewise, the OXPHOS pathway was in the top 10 significantly enriched pathways with a predicted upregulation (z-score: 3.2) (Fig. 4a); individual OXPHOS proteins also revealed a consistent upregulation (Fig. 4b). Other top 10 pathways were related to ethanol and metabolite degradation. Additionally, 57 of these differentially expressed proteins associated with genes were also significantly differentially regulated at the transcriptional level in the KOBS cohort (Supplemental Table 9 (32)).

### Association of Mitochondria-Related Gene Expression With Clinical Characteristics

Finally, following the transcriptome analyses, we studied the relationship between SAT mitochondria-related gene expression and improvements in clinical characteristics following weight loss. Following the 8-month DiOGenes intervention, the change in the combined score for 102 genes in the OXPHOS pathway (OXPHOS score) negatively associated with LDL cholesterol and positively with HDL cholesterol as well as improvements in insulin sensitivity measures (Supplemental Table 10, model 1 (32)). Similar associations were observed for HDL in KOBS. Following further adjustments for age and sex (Supplemental Table 10, model 2 (32)), only the association between the OXPHOS score and HDL remained significant. These results imply that a relative increase in the OXPHOS pathway following weight loss may associate with improved metabolic outcomes.

We also analyzed the associations between the OXPHOS pathway and clinical parameters using a dimension reduction by PCA. PC1 (composed of 57 genes, Supplemental Table 11 (32)) and PC3 (24 genes) significantly associated with changes in HDL, total cholesterol, and fasting insulin in the DiOGenes cohort (Supplemental Table 10, model 3 (32)). In the KOBS cohort, PC2 (22 genes) associated with HDL and fasting glucose (Supplemental Table 10, model 3 (32)). Interestingly, all mitochondria-encoded genes clustered together in the same PC (PC3 in DiOGenes and PC2 in KOBS, Supplemental Table 11 (32)). Overall, these associations suggest a relationship between changes in OXPHOS transcripts and clinical improvements following weight loss in both interventions.

## Discussion

In this study, we investigated how the SAT transcriptional capacity for mitochondria changed following diet- and surgery-induced interventions in 4 independent, pan-European weight-loss studies using a standardized set of bioinformatics analyses. Our findings show that mitochondria-related gene expression is regulated in opposing directions following diet-induced vs surgically induced weight loss with a long-term follow-up. Specifically, diet-induced weight loss downregulated, while surgery-induced weight loss upregulated mitochondrial oxidative metabolism (based on global transcriptomics and pathway analyses). We observed the strongest effects in mtDNA-encoded mRNA transcripts. Additionally, we confirmed upregulated mitochondrial oxidative metabolism at the protein level following surgery-induced weight loss.

The finding that diet-induced weight loss leads to an apparent transcriptional downregulation of SAT oxidative metabolism is intriguing and slightly counterintuitive, since adipose tissue is known to be a low oxygen-consuming tissue (33). Previously, we showed that mitochondrial density and OXPHOS complex III protein levels decreased, particularly in individuals who continuously lost weight following the 12-month dieting; yet, we observed no effects on other OXPHOS proteins or mtDNA transcripts (12). The downregulation of oxidative pathways may contribute to or be a consequence of other cellular disruptions, such as those related to lipid metabolism, energy partitioning and oxidative stress response following weight loss (13, 25). On a more functional level, one study reported indirect evidence for SAT mitochondria changes following diet-induced weight loss through a reduction in the SAT oxygen pressure (34). However, SAT mitochondrial functional alterations remain to be studied to elucidate biological mechanisms since no studies have directly measured mitochondrial respiration from fresh SAT biopsies following diet-induced weight loss.

After bariatric surgery, we observed a consistent and significant upregulation of the OXPHOS pathway in SAT. In the short-term weight-loss response, within 1 week to 3 months, an increased expression of the genes involved in mitochondrial biogenesis was observed following gastric bypass surgery (but not gastric banding) (16). Furthermore, the SAT expression of OXPHOS genes appeared to increase at 3 months following gastric bypass (35). In the longer term, at the 1-year postoperative time point, relatively small studies demonstrated that gastric bypass surgery increased the SAT gene expression in the mitochondrial metabolic and OXPHOS pathways (18, 36). Our results here confirm and extend these findings, since our weight-loss studies included more individuals (KOBS) and longer follow-up periods (DEOSH). Notably, our proteomics results further confirm the increased OXPHOS findings at the posttranscriptional level. The beneficial effects of bariatric surgery on SAT mitochondrial oxidative metabolism may be mediated by multiple signals. Bile acids (37), gut hormones (38), fibroblast growth factors 19 and 21 (39), and microbiome-related products including short-chain fatty acids (40) represent the most promising candidates to explain such changes since their levels are altered to a greater degree after bariatric surgery compared with diet-induced weight loss. Furthermore, surgery-induced weight loss also leads to a greater reduction in adipocyte size when compared with diet-induced weight loss. This may positively influence the mitochondrial oxidative metabolism given that a relatively greater hyperplasia follows surgery (23).

Since the transcriptional capacity of mitochondria changed following weight loss, we hypothesized that this would affect SAT function and thus whole-body physiology. Therefore, we studied the relationship between OXPHOS gene expression in SAT and improvements to clinical characteristics following weight loss. We found similar associations between the OXPHOS score and the mitochondria-encoded genes with insulin sensitivity–related measures in the DiOGenes and KOBS cohorts. Nevertheless, the small effect sizes in our results also indicate that diet- and surgery-induced weight loss result in clinical improvements through mechanisms not directly dependent on the transcriptional capacity of SAT mitochondria.

The primary strength of this study lies in our reliance on 4 independent weight-loss interventions: the large DiOGenes (diet-induced) and KOBS (surgery-induced) studies for our primary analyses, and the smaller CRYO and DEOSH studies to replicate the findings. Importantly, we used weight-loss percentage as the primary explanatory variable, adjusted for sex and age, thereby accounting for differences in the degree of weight loss achieved following intervention. We confirmed our findings in the matched 15% to 20% weight-loss groups in DiOGenes and KOBS. Although, at the time of the biopsies, the energy balance of the study participants could have varied, it is likely that on average a stable weight had been achieved at the long-term timepoints. Additionally, while our results primarily stem from transcriptomics, we could validate our findings at the protein level in the surgery-induced KOBS study. Follow-up studies should examine the functional consequences of long-term weight loss on SAT mitochondrial activity and function, since posttranscriptional and/or translational regulatory mechanisms could also be affected.

In conclusion, this study shows that long-term diet-induced weight loss transcriptionally downregulated mitochondrial pathways in SAT. We also identified an opposite pattern among long-term weight loss induced by bariatric surgery supported by protein-level data. These data open new and exciting avenues for utilizing molecular mechanisms in SAT affected by different types of weight-loss interventions, possibly leading to improved tailored weight-loss interventions. While both weight-loss methods induce impressive benefits on metabolic health, dieting carries a significantly higher likelihood for long-term weight regain. Whether this propensity relates to the selective downregulation of mitochondrial oxidative metabolism in SAT requires clarity. Future studies are warranted to obtain detailed insight into mitochondrial function in SAT as a potential target for weight-loss success.

## Data Availability

Some or all datasets generated during and/or analyzed during the current study are not publicly available but are available from the corresponding author on reasonable request. ***Resource Availability:*** Transcriptomics data are made available from the Gene Expression Omnibus under accession no. GSE95640 for DiOGenes (25) and no. GSE103769 for CRYO (12). Other data are unsuitable for public deposition due to ethical restrictions and the privacy of participant information.

## Abbreviations

BCAA: branched-chain amino acid
BMI: body mass index
FDR: false discovery rate
IPA: ingenuity pathway analysis
LCD: low-calorie diet
LC-ESI-MS/MS: liquid chromatography electrospray ionization tandem mass spectrometry
mtDNA: mitochondrial DNA
OXPHOS: oxidative phosphorylation
PC1-3: principal components 1-3
PCA: principal component analysis
RYGB: Roux-en-Y gastric bypass
SAT: subcutaneous adipose tissue
T2DM: type 2 diabetes mellitus
TCA: tricarboxylic acid (cycle)

## Additional Information

***Disclosures:*** At the time of the study, A.V. was a full-time employee at Nestlé Institute of Health Sciences SA. W.H.M.S. discloses the receipt of research support from several food companies, such as Nestlé, DSM, Unilever, Nutrition et Sante and Danone as well as pharmaceutical companies including GSK, Novartis, and Novo Nordisk; he is also an unpaid scientific advisor for the International Life Science Institute (ILSI) Europe. A.A. discloses grants and personal fees received from Gelesis, USA; personal fees from Acino, Switzerland; BioCare Copenhagen, DK; Dutch Beer Institute, NL; Groupe Éthique et Santé, France; IKEA Food Scientific Health Advisory Board, SE; McCain Foods Limited, USA; Navamedic, DK; Novo Nordisk, DK; Pfizer, USA; Saniona, DK; Weight Watchers, USA & Zaluvida, Switzerland; and grants from DC-Ingredients Denmark, which lie beyond the scope of the work reported here. E.E.B. received financial support from food industry, such as DSM, Danone, Friesland Campina, Avebe, and Sensus, partly within the context of public-private consortia and has received funding from pharmaceutical companies, including Novartis. She is involved in several task forces and expert groups related to ILSI Europe.
